# Role of metabolic reprogramming in pro-inflammatory cytokine secretion from LPS or silica-activated macrophages

**DOI:** 10.3389/fimmu.2022.936167

**Published:** 2022-10-21

**Authors:** Antonella Marrocco, Luis A. Ortiz

**Affiliations:** ^1^ Department of Environmental and Occupational Health, School of Public Health, University of Pittsburgh, Pittsburgh, PA, United States; ^2^ Department of Environmental Health, Harvard T.H. Chan School of Public Health, Boston, MA, United States

**Keywords:** macrophage metabolic adaptation, macrophage immunometabolism, M1 macrophages, respirable crystalline silica, complex II, electron transport chain, mitochondria

## Abstract

In the lungs, macrophages constitute the first line of defense against pathogens and foreign bodies and play a fundamental role in maintaining tissue homeostasis. Activated macrophages show altered immunometabolism and metabolic changes governing immune effector mechanisms, such as cytokine secretion characterizing their classic (M1) or alternative (M2) activation. Lipopolysaccharide (LPS)-stimulated macrophages demonstrate enhanced glycolysis, blocked succinate dehydrogenase (SDH), and increased secretion of interleukin-1 beta (IL-1β) and tumor necrosis factor-alpha (TNF-α). Glycolysis suppression using 2 deoxyglucose in LPS-stimulated macrophages inhibits IL-1β secretion, but not TNF-α, indicating metabolic pathway specificity that determines cytokine production. In contrast to LPS, the nature of the immunometabolic responses induced by non-organic particles, such as silica, in macrophages, its contribution to cytokine specification, and disease pathogenesis are not well understood. Silica-stimulated macrophages activate pattern recognition receptors (PRRs) and NLRP3 inflammasome and release IL-1β, TNF-α, and interferons, which are the key mediators of silicosis pathogenesis. In contrast to bacteria, silica particles cannot be degraded, and the persistent macrophage activation results in an increased NADPH oxidase (Phox) activation and mitochondrial reactive oxygen species (ROS) production, ultimately leading to macrophage death and release of silica particles that perpetuate inflammation. In this manuscript, we reviewed the effects of silica on macrophage mitochondrial respiration and central carbon metabolism determining cytokine specification responsible for the sustained inflammatory responses in the lungs.

## 1 Introduction

In 1927, Otto Warburg discovered the so-called Warburg effect, which portrays the ability of tumor cells to reprogram their metabolism to survive through the upregulation of glycolysis and suppression of oxidative phosphorylation (OXPHOS) in the presence of abundant oxygen (1). Since then, our understanding of immunometabolism, the metabolic signatures of immune cells, and the adaptation of metabolic pathways governing the molecular transcriptional and post-transcriptional mechanisms of immune cells in response to stimuli have expanded tremendously (2–5). In the lungs, macrophages constitute the frontline cells of innate immunity against pathogens and foreign bodies and play a key role in the maintenance of tissue homeostasis, resolution of inflammation, and tissue regeneration/repair after injury (6, 7). Thus, the stimuli released in the surrounding microenvironment can determine the profile of macrophages, switching from the aerobic OXPHOS-driven to the anaerobic glycolysis-driven phenotype and vice-versa. These different metabolic profiles are associated with the release of distinct and diverse mediators (pro- and anti-inflammatory cytokines) whose specification is highly regulated by the cellular metabolic signatures.

In this review, the authors described how an immune response of macrophages, particularly alveolar macrophages, undergoes their metabolic adaptation leading to functional polarization. To accomplish this goal, we described the difference between the mediators released by macrophages in response to lipopolysaccharide (LPS) versus those observed following the activation of macrophages by airborne crystalline silica.

## 2 Activation of macrophages

**Figure 1:**
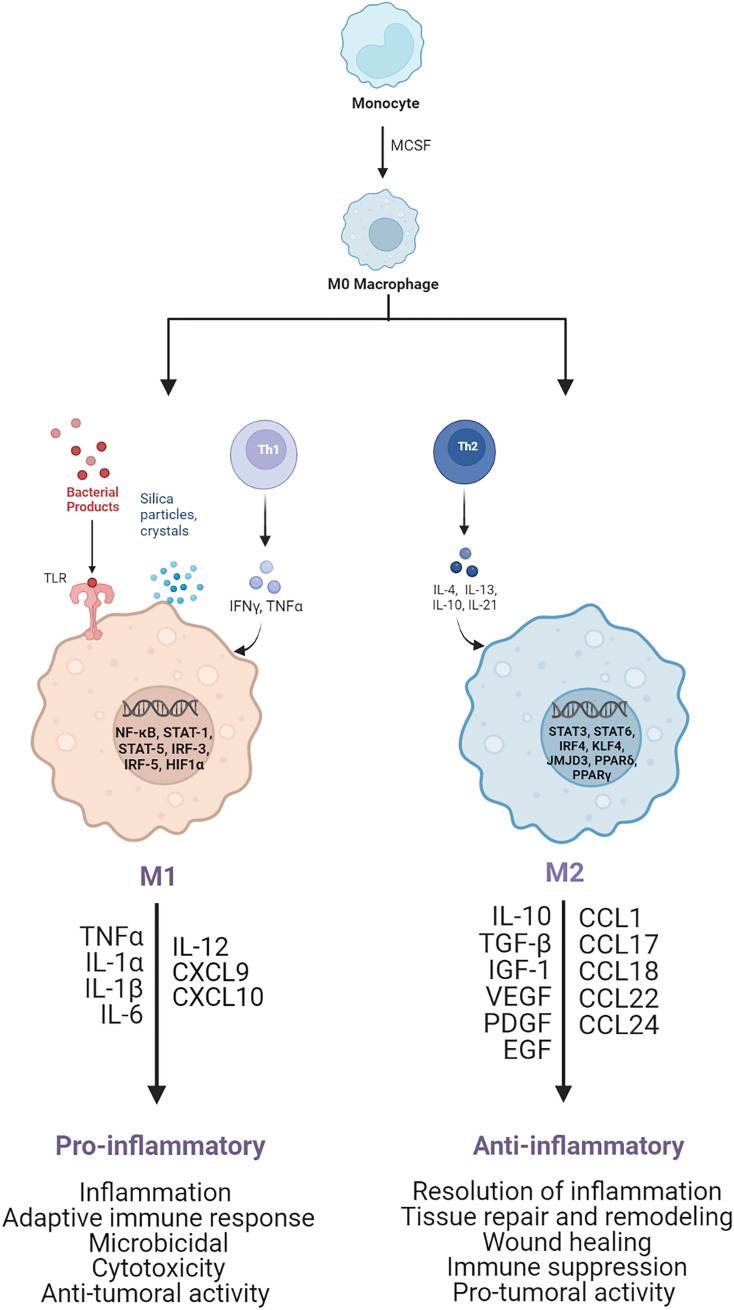
Schematic of M1 and M2 macrophage activation. M1 pro-inflammatory macrophages are activated by TLRs ligands, such as LPS, or Th1 cytokines, such as TNF-α and IFN-γ. After activation, several transcription factors are involved, such as NF-kB, STAT1, STAT5, IRF3, and IRF5, leading to the release of pro-inflammatory cytokines and chemoxines, including TNF-α, IL-1α, IL-1β, IL-6, IL-12, CXCL9, and CXCL10, which exert microbicidal and anti-tumoral functions. M2 anti-inflammatory macrophages are polarized by Th2 cytokines, such as IL-4 and IL-13, which activate transcription factors, including STAT3, STAT6, IRF4, KLF4, JMJD3, PPARδ, PPARγ. As result the M2 activated macrophages release anti-inflammatory cytokines and chemoxines including IL-10, TGF-β, CCL17, CCL18, and CCL22, which promote wound healing, tissue repair and regeneration, immune-suppression and tumor grow and diffusion.

Macrophages are very plastic cells, capable of changing and adapting their phenotype based on the surrounding environmental stimuli and according to their functional requirement (8, 9). During the early stages of the immune response, macrophages recognize and ingest pathogens and foreign bodies, undergoing activation (10). During the later stages, activated macrophages are responsible for the resolution of inflammation, fibroproliferative response regulation, wound healing, and tissue repair (11–14). Based on the interaction with specific stimuli and the following gene expression, transcriptional and post-transcriptional regulation, and mediator secretion, two main activated profiles of macrophages have been recognized: classically activated pro-inflammatory M1 macrophages (15–19) and alternatively activated anti-inflammatory M2 macrophages (11, 17–22), as shown in **Figure 1**.

Generally, M1 pro-inflammatory macrophages are triggered by the detection of danger signals through the intracellular pattern-recognition receptors (PRR) and toll-like receptors (TLRs). Specifically, the TLR-ligands (e.g. LPS) and T helper-1 lymphocytes-secreted cytokines, such as interferon-γ (IFN-γ) and tumor necrosis factor-alpha (TNF-α), can activate macrophages to sustain the immune response and confer a higher host defense.

The effect of LPS on macrophages promotes the activation of transcription factors, such as nuclear factor kappa-light-chain enhancer of B-cell (NF-κB) (22–26), signal transducer and activator of transcription molecules (STAT1/3) (23–25), hypoxia-induced factor (HIF)-1α (27–30), and activator protein (AP)-1 (25). This transcriptional activation leads to the secretion of high levels of pro-inflammatory cytokines, such as TNF-α, interleukin (IL)-1β, IL-6, IL-12, and IL-23, as well as reactive oxygen species (ROS) and inducible nitric oxide synthase (iNOS) (31, 32), which are characteristic of the M1 polarization and are necessary to sustain the inflammatory reaction and recruit other immune cells to initiate the adaptive immune response. Nevertheless, these cytokines are also cytotoxic; hence, their regulation is extremely crucial to avoid further tissue damage. Specifically LPS-activated macrophages also synthesize eicosanoids, such as prostaglandin E_2_ (PGE_2_), which are bioactive lipids capable of autoregulate the inflammation, through downregulation of pro-inflammatory cytokines (i.e., TNF-α) and upregulation of anti-inflammatory cytokines (i.e., IL-10).

M2 anti-inflammatory macrophages differentiate in response to innate or adaptive signals. IL-4 (19, 33, 34) or IL-13 (19, 33, 34), released by mast cells, basophils, and T helper-2 lymphocytes, promotes macrophages differentiation into cells involved in inflammation resolution, tissue repair and remodeling, wound healing, immune suppression, and pro-tumoral activity (9, 19, 34–36). Typical M2 activated macrophages surface markers are the mannose receptor (CD206), the decoy IL-1R (receptor), and IL-1R antagonist, while released biomarkers are anti-inflammatory and pro-fibrotic cytokines, such as the transforming growth factor-beta (TGF-β) and insulin-like growth factor 1 (IGF-1) (9, 35, 37, 38), and pro-angiogenetic factors, such as vascular endothelial growth factor A (VEGF-A), endothelial growth factor (EGF), platelet-derived growth factor (PDGF), and IL-8 (35, 39, 40). The predominant molecular pathways involved in the cytokines specification of M2 macrophages include STAT, GATA binding protein 3 (GATA3), suppressor of cytokine signaling 1 (SOCS1), peroxisome proliferator-activated receptor-gamma (PPARγ) (26, 34, 37, 41) **(** **Figure 1** **)**.

Within the M2-activated macrophages, there are other populations of cells, namely regulatory macrophages, which can be activated by various factors. Specifically, M2a macrophages are activated by IL4/13 leading to increased expression of IL-10, TGF-β, CCL17, CCL18, and CCL22. M2b macrophages are promoted by immune complexes/TLR-ligands/IL1b and are characterized by increased secretion of both pro- and anti-inflammatory cytokines, such as TNF-α, IL-1β, IL-6, and IL-10. M2c macrophages are triggered by glucocorticoids/IL10/TGFβ and subsequently release IL-10, TGF-β, CCL16, and CCL18. In addition, in the tumor microenvironment there are M2d macrophages, which are activated by TLR antagonists, and secrete IL-10 and vascular endothelial growth factors (VEGF), which are involved in angiogenesis and tumor progression (42–45). These regulatory cells can produce both pro- and anti-inflammatory cytokines, through the activation of transcription factors, such as STAT6, interferon regulatory factor 4 (IRF4), NF-kB, and PPAR-γ. The main purpose of these macrophages is to regulate the immune and inflammatory response, wound healing, angiogenesis, and tumor growth and diffusion (9, 46–48).

## 3 Metabolic features of LPS-activated macrophages

**Figure 2:**
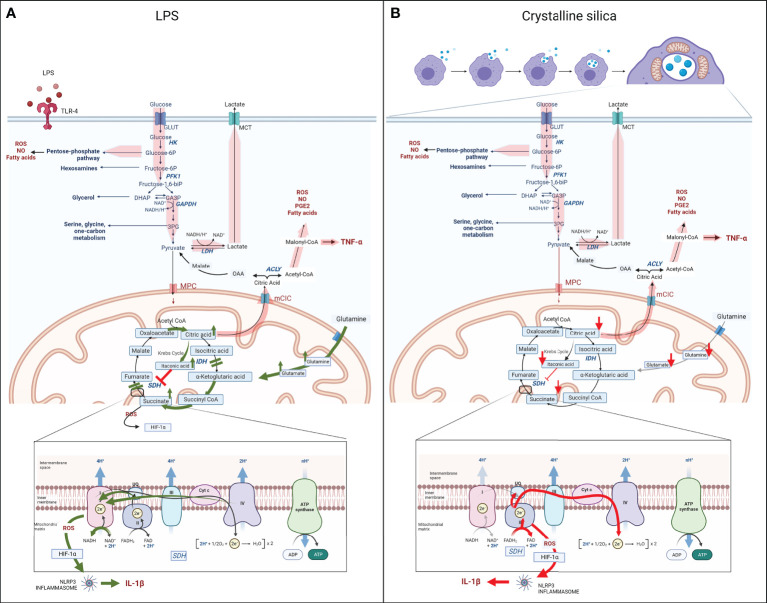
Schematic of the metabolic reprogramming of M1 macrophages following LPS **(A)** or silica **(B)** exposure. Upon LPS or silica activation, M1 macrophages show enhancement of aerobic glycolysis, glucose uptake, and conversion into lactate; increased flux through the PPP, NADPH generation, and production of fatty acids, NO, and ROS (wide red arrows). LPS induces two breakpoints in the Krebs cycle (IDH and SDH), leading to the mitochondrial accumulation of citrate and succinate. Accumulation of citrate increases the synthesis of itaconate, and citrate translocation into the cytosol, *via* mCIC, resulting in upregulation of synthesis of fatty acids, PGE2, NO, ROS, and transcription and secretion of TNF-α (wide red arrows). The dysfunction of SDH (Complex II of ETC), results in the accumulation of succinate, also due to the glutamine-dependent anaplerosis *via* the GABA shunt, and reversion of the electron transport (RET) toward complex I, which rises the generation of ROS, stabilization of HIF-1α, activation of the NLRP3 inflammasome and release of pro-inflammatory cytokine IL-1β. (LPS-specific activated pathways are represented with green arrows.) In contrast to LPS, silica also modulates the Krebs cycle, but the intracellular level of all key Krebs cycle intermediates, including succinate and citrate, and amino acids is measured below baseline, probably as a result of high demand and consumption (red arrows). In addition, the enhanced aerobic glycolysis occurs at the expense of mitochondrial respiration, which is sustained only by an upregulated complex II activity, while complex I is impaired, leading to the generation of an excessive amount of ROS. The latter in addition to the intrinsic toxicity of silica particles drive the stabilization of HIF-1α, activation of NRLP3 inflammasome, and release of IL-1β. Similar to LPS-activated macrophages, hyperproduction of malonyl-CoA is necessary for the synthesis of fatty acids, PGE2, NO, ROS, and transcription and secretion of TNF-α (wide red arrows). (Silica-specific activated pathways are represented with red arrows.).

As shown in **Figure 2A**, the main metabolic features of LPS-activated macrophages are represented by enhanced flux through glycolysis, pentose phosphate pathway (PPP), and fatty acid synthesis (FAS) at the expense of the mitochondria respiration and Krebs cycle, where the impairment of isocitrate dehydrogenase (IDH) and succinate dehydrogenase (SDH) causes the intracellular accumulation of citrate and succinate (49–57). Altogether, these metabolic alterations drive the pro-inflammatory status and the transcription and secretion of pro-inflammatory mediators, such as IL-1β, TNF-α, and IFNs. In contrast, M2 anti-inflammatory macrophages are metabolically sustained by mitochondrial respiration, while glycolysis and PPP are decreased, and upregulated fatty acid oxidation (FAO), glutaminolysis, and tryptophan catabolism with the release of kynurenine and synthesis of polyamines (49, 58).

### 3.1 Glycolysis and the pentose phosphate pathway (PPP)

Although upregulated glycolysis in pro-inflammatory macrophages was recognized early during the 1900s (1, 59), the link between this metabolic reprogramming process, the production and release of inflammatory cytokines, and macrophage polarization has only been recently recognized (3, 5).

A crucial role of LPS-activated macrophages in the metabolic and functional regulation of immune response is mediated by HIF-1α (27, 29, 30, 60–62). HIF-1α regulates the main glycolytic alterations, such as overexpression of glucose transporter 1 (GLUT1), which facilitates rapid and increased uptake of glucose (54, 63, 64), and overactivation of pyruvate dehydrogenase kinase isozyme 1 (PDK1) and lactate dehydrogenase (LDH), which prevent pyruvate from entering into the Krebs cycle in favor to its conversion into lactate in a reaction coupled with the oxidation of nicotinamide adenine dinucleotide (NADH) into NAD^+^ (46, 65–67) **(** **Figure 2A** **)**. The latter redox reaction is particularly relevant for the enhancement of the glycolytic flux, occurring in cooperation with the overexpression of other key enzymes, such as 6-phosphofructo-2-kinase/fructose-2,6-bisphosphatase 3 (PFKFB-3), pyruvate kinase M2 isoform (PKM2), and monocarboxylate transporter 4 (MCT4), which are also regulated by HIF-1α (68–70).

Interestingly, evidence has shown a correlation between the pro-glycolytic metabolic alteration and inflammasome activation (52, 70–73). LPS treatment of peritoneal macrophages induces mTORC upregulation, associated with hyperexpression of glycolytic enzymes, such as hexokinase (HK)-1, and simultaneous overactivation of the NLR family pyrin domain containing 3 (NLRP3) inflammasome (70–72, 74, 75). Moreover, inhibition of glycolysis with 2-deoxyglucose (2DG) in LPS-activated bone marrow-derived macrophages (BMDM) and an *in vivo* model of murine acute lung injury has suppressed the secretion of the activated NLRP3 inflammasome product, IL-1β, while it did not affect TNF-α or IL-6 secretion (48, 52, 70, 73). In an association with the glycolytic pathway, the upregulation of PPP in pro-inflammatory macrophages provides the reduced NADPH (nicotinamide adenine dinucleotide phosphate) from NADP^+^, which is required as a cofactor for LPS-activated iNOS to catabolize arginine into nitric oxide (NO) and L-citrulline (73, 76, 77), but it also enhances the fatty acid synthesis, required for prostaglandin production (24, 48, 49, 73, 78, 79).

### 3.2 Krebs cycle

Metabolomic analysis of LPS-activated macrophages has revealed two important breakpoints in the Krebs cycle, represented by the downregulation of IDH, which converts the citrate isomer isocitrate into α-ketoglutarate, and SDH, which converts succinate into fumarate (62, 73, 80, 81). The direct consequence of these changes is the intracellular accumulation of substrates for both enzymes, citrate and succinate (62, 80–83).

Citrate is necessary for the production of three important pro-inflammatory mediators: NO and ROS through the reduction of NADP^+^ to NADPH and PGE_2_ due to the upregulation of mitochondria citrate carrier (mCIC) (80, 84). In LPS-activated macrophages, the mCIC overexpression allows for the translocation of citrate from mitochondria into the cytosol, where it is cleaved to acetyl-CoA and oxaloacetate by the upregulated enzyme adenine triphosphate (ATP) -citrate lyase (ACLY). While in the cytosol, acetyl-CoA represents the substrate for FAS and, in turn, phospholipids and arachidonic acid, which are the precursors of PGE2 (80, 82, 85–88) **(** **Figure 2A** **)**.

More recently, investigations on the citrate-derived FAS-precursors have revealed that in LPS-activated macrophages, malonyl-CoA induces the malonylation of several proteins, including glycolytic enzyme glyceraldehyde-3-phosphate dehydrogenase (GAPDH). In resting macrophages, GAPDH sequesters TNF-α mRNA, blocking its translation, while the LPS-induced malonylation of GAPDH facilitates the release of TNF-α mRNA for transcription and subsequent protein secretion (62, 89).

Additionally, the accumulation of citrate in mitochondria of M1 macrophages also leads to the upregulation of enzyme aconitate decarboxylase 1 (ACOD1), also known as immune-responsive gene 1 protein (Irg1), that converts aconitate (derived from citrate) to itaconic acid, leading to the accumulation of the latter (81, 90–93) **(** **Figure 2A** **)**.

In addition to antimicrobial effects against gram-positive and gram-negative bacteria, such as *Legionella pneumophila*, *Mycobacterium tuberculosis*, and *Salmonella enterica* (94–96), itaconate plays a crucial role in the immunomodulation and suppression of inflammatory response by LPS-activated macrophages due to the stabilization of anti-inflammatory transcription factor nuclear factor erythroid 2-related factor 2 (NRF2) *via* the Kelch-like ECH-associated protein 1 (KEAP1) degradation. The subsequent NRF2 nuclear translocation leads to the reduction of ROS production, inflammasome activation, and pro-inflammatory cytokines secretion, such as IL-1β and IL-6 (67, 81, 92, 93, 97–102).

Additionally, itaconic acid inhibits SDH, causing succinate accumulation (46, 50, 52, 53, 62, 82, 83, 101, 103, 104) **(** **Figure 2A** **)**. SDH has the unique property of being an enzyme shared between the Krebs cycle and the mitochondrial electron transport chain (ETC), where it represents respiratory complex II. For this particular feature, SDH catalyzes the oxidation of succinate to fumarate by coupling it with the reduction of ubiquinone (UQ) to ubiquinol (UQH2) and FAD (flavin adenine dinucleotide) to FADH_2_. These reactions are fundamental in the ATP generation process occurring in the ETC complex V or ATPase enzyme. In LPS-activated macrophages, the increased concentration of succinate (complex II substrate) leads to the over-reduction of ubiquinone to ubiquinol; thus, the electrons are transferred back to respiratory complex I in a process known as reverse electron transport (RET) (105–107). In turn, RET is responsible for significant ROS generation that, along with the succinate cytosol accumulation, drives the stabilization of HIF-1α by inhibiting prolyl hydroxylase domain (PHD) activity (46, 61, 66, 86, 108–112), activation of the NLRP3 inflammasome, and release of mature IL-1β (97, 100, 113–115) **(** **Figure 2A** **)**. Subsequently, LPS treatment promotes the overexpression of succinate receptor SUCNR1/GPR91, helping sustain the inflammation and the IL-1β secretion by sensing succinate in the extracellular space (104).

Several mechanisms explain the carbon flow through the dysfunctional Krebs cycle of LPS-stimulated macrophages. These predominantly involve the metabolism of the amino acids, which can contribute to sustaining the amount of α-ketoglutarate, succinate, and fumarate, even in the presence of enzyme inhibition (46, 116). In M1 macrophages, the upregulation of aspartate-arginosuccinate shunt (AASS), which is primarily involved in the iNOS expression and antimicrobial NO and IL-6 production, also represents the source of fumarate and, consequently, citrate after SDH inhibition by anaplerosis (117). Similarly, the glutamine-dependent anaplerosis *via* the gamma-aminobutyric acid (GABA) shunt represents an independent source of succinate, in which α-ketoglutarate or glutamine is converted into glutamate, contributing to the increased concentration of succinate in LPS-activated macrophages (118, 119). This is also supported by the increased expression of glutamine transporter Slc3a2 in LPS-activated macrophages (52, 120).

### 3.3 Electron transport chain

The aforementioned overproduction of NO from arginine in LPS-activated macrophages plays a crucial role in the dysfunctional mitochondrial respiration: nitrosylation of the iron-sulfur-containing ETC complexes I, II, and IV inhibits mitochondrial respiration (121–123), uncouples the electron transport causing leakage of electrons, decreasing ATP production, and promoting ROS generation mainly by the RET at complex I (51, 115, 122).

Complex I and II are highly involved in macrophageal immune response against bacteria. Recent investigation on the role of ECSIT protein (evolutionarily conserved signaling intermediate in Toll pathways) has revealed that besides being a key regulator in complex I assembly, upon bacteria phagocytosis, ECSIT initiates the recruitment of mitochondria into the phagosome to produce and secrete ROS and other antibacterial and pro-inflammatory mediators (IL-6, TNF-α, and IL-1β) into the phagosomes (124).

Similarly, Garaude et al. have reported that after the engulfment of live bacteria, BMDMs recruit mitochondria into the phagosome to produce toxic products, such as ROS, fumarate, itaconic acid, and others, that are secreted into the phagosomes. Such metabolically adapted mitochondria exhibited an alteration in the assembly of ETC supercomplexes, consisting of reduced complex I activity and enhanced complex II abundance and activity (125, 126). Nonetheless, the same phenomenon has not been present in LPS-activated BMDM.

In summary, LPS-activated macrophages are metabolically sustained by increased glucose uptake, glycolysis rate, and FAS supplying the source of energy in the absence of an efficient mitochondria respiration due to ETC and oxidative phosphorylation suppression and impaired Krebs cycle. These metabolic adaptations of macrophages govern the immune response, bacterial killing, and host defense through the release of toxic and pro-inflammatory mediators, such as ROS, NO, IL-6, TNF-α, and IL-1β.

## 4 Metabolic reprogramming of macrophages in silica-induced pulmonary fibrosis

Airborne crystalline silica, also known as silicon dioxide (SiO_2_), is one of the most abundant minerals on Earth. More than 2 million workers in the United States and more than 230 million workers worldwide are exposed to crystalline silica annually, predominantly due to activities, such as mining and construction (127, 128). The health effects of silica inhalation are mainly characterized by chronic lung inflammation and progressive fibrosis. Therefore, silicosis is considered progressive pneumoconiosis without any specific and effective available therapy and is associated with an increased risk of tuberculosis, lung cancer, chronic obstructive pulmonary disease, kidney disease, and autoimmune disease, even after ceased silica exposure (129–132).

The understanding of silicosis pathogenesis is still incomplete, and almost no data exist in humans (133, 134). Previous studies have shown that alveolar macrophages are the first line of defense against crystalline silica and play a crucial role in initiating lung inflammation (129, 135–137). Investigation on alveolar macrophages from humans exposed to asbestos or silica has shown a spontaneous release of IL-1β from activated cells (62, 138), which was subsequently related to NLRP3 inflammasome activation (138–141).

The initial response to crystalline silica in the lungs is mediated by innate immunity. Phagocytosis of silica particles by macrophages into phagosomes triggers macrophageal activation at the cellular and molecular level with the development of inflammatory response through oxidative stress, the release of ROS, and activation of PRRs and NLRP3 inflammasome, followed by transcription and secretion of inflammatory cytokines, such as IL-1β, TNFα, and IFNs (133, 135, 136, 139, 141–145). Since silica particles cannot be degraded, the persistent macrophageal activation results in increased NADPH oxidase (Phox) activity and ROS production, with eventual cell death (apoptosis/necrosis) and release of silica particles perpetuating and amplifying inflammation (62, 129, 146–152).

However, it is important to mention that lung macrophages polarization is a dynamic process depending on the stage of silicosis and the ontogenesis of macrophages (153–155). Indeed, in an early acute and chronic inflammatory stage, the resident and recruited alveolar macrophages are predominant and exhibit mostly an M1 pro-inflammatory phenotype (increased glycolysis rate and impaired mitochondrial respiration and Krebs cycle enzymes) accompanied by high expression of inflammatory cytokines, such as TNF-α, IL-1β, IL-6, ROS, and iNOS, which are necessary to eliminate the pathogen. Subsequently, in the late fibrosis stage, the resident interstitial macrophages are mostly involved in tissue repair, remodeling, and fibrogenesis, and they are characterized by an M2 anti-inflammatory phenotype (even when stimulated with LPS, they maintain stable or minimally altered glycolysis and Krebs cycle enzymes and increased fatty acid oxidation even after LPS stimulation) accompanied by high expression of anti-inflammatory cytokines, such as IL-10, PGE2, and TGF-β (153–158).

However, while there are studies explaining the many different aspects of mitochondrial reprogramming induced by LPS, very little data are available regarding the correlation between metabolic features and cytokines secretion from M1 macrophages during the early acute response to crystalline silica.

### 4.1 Immunometabolic response of macrophages to crystalline silica

#### 4.1.1 Glycolysis

Recent *in vivo* and *in vitro* studies have shown that in response to silica, without LPS-priming, lung macrophages could adapt their metabolism, raising the flux through aerobic glycolysis, which becomes a major source of ATP (62, 159–162). Similar to the LPS-activated macrophages, the levels of released lactate and glycolytic enzymes expression, including HK2, PKM2, PDK1, and LDH, have been all enhanced in macrophages of silicotic rodents lungs (151, 161–163) and murine macrophages cell lines (62) (**Figure 2B** **)**. However, the increased release of lactate has been further enhanced *in vitro* by LPS-priming of macrophages before silica exposure, reflecting the surge of LDH release and cell necrosis in these experimental conditions (62, 164, 165).

#### 4.1.2 Krebs cycle

While data on Krebs cycle alteration following silica exposure in macrophages *in vivo* have been not available, recent reports have shown clear differences between the LPS- and silica-activated RAW 264.7 macrophages in the functionality of the Krebs cycle, as represented in **Figure 2B**. In contrast to the LPS-activated macrophages, which recapitulate all above-mentioned features, including an increased level of itaconate and succinate correlated to the impairments of IDH and SDH enzymes **(** **Figure 2A** **)**, silica-treated RAW 264.7 macrophages exhibit an overall intracellular depletion of Krebs cycle metabolites, including itaconate, α-ketoglutarate, succinate, fumarate, and malate, and undetectable citrate, accompanied by the depletion of amino acids, such as glutamine and glutamate, in the absence of the GABA shunt activation (62) **(** **Figure 2B** **)**.

#### 4.1.3 Electron transport chain

The assessment of mitochondrial respiration in LPS- and silica-activated RAW 264.7 macrophages has also revealed profound differences. While LPS has not affected complex I activity but has only slightly enhanced complex II activity (125), crystalline silica alone has been capable of enhancing the oxygen flux through complex II, even after complex I inhibition with rotenone, which proves that RET has not been the reason. More importantly, the overactivation of complex II has been linked to the decreased activity of complex I, most probably due to ECSIT downregulation and a surge in ROS production (62) **(** **Figure 2B** **)**. The crucial role of complex II in macrophageal immune response and survival has been further elucidated by using the IC-21 mouse macrophage cell line. IC-21 macrophages exposed to crystalline silica in the absence of LPS-priming have experienced a high cell death, which is correlated with the downregulation of complex II activity, and the increased oxygen flux and mROS generation through complex II when stimulated with CII substrate succinate (62). Similar to macrophages, *in vitro* studies on human bronchial epithelial (HBE) cells have indicated that crystalline silica alone also determines a mitochondrial membrane depolarization depending on NLRP3 phosphorylation and activation (166).

#### 4.1.4 Correlation between metabolic alteration and cytokine secretion

While it is evident that silica-activated macrophages secrete IL-1β, TNF-α, IFNs, IL-6, and ROS to initiate a strong inflammatory response, the silica-driven metabolic alterations, their related transcription, and translation effects are still unclear.

Similar to LPS, silica-activated macrophages exhibit a stabilization of HIF-1α and HIF-1α-mediated activation of NRLP3 inflammasome, leading to the release of IL-1β. However, while LPS promotes the stabilization of HIF-1α through the impairment of the Krebs cycle and accumulation of succinate, in silica-activated macrophages lacking a high level of succinate, the excessive amount of ROS and the toxicity of silica particles sustain the stabilization of HIF-1α and subsequent NRLP3 inflammasome activation, followed by IL-1β cleavage and secretion (62) **(** **Figure 2B** **)**.

In both LPS- and silica-activated RAW 264.7 macrophages, the malonylation of GAPDH facilitates the release of TNF-α mRNA for transcription and secretion (62). Interestingly, the secretion of TNF- α, which is a key mediator of silicosis, is not a common feature of silica-activated macrophages. In fact, while LPS-activated IC-21 and RAW 264.7 macrophages secrete high levels of TNF-α following NF-κB activation, only RAW 264.7 macrophages maintain the enhanced TNF-α production and NF-κB activation in response to silica, which is absent in IC-21 macrophages, although both can phagocytize silica particles (130, 146, 150, 152, 167).

Finally, the decreased intracellular level of itaconate in silica-activated macrophages correlates with the decreased transcription and secretion of IFN-β, as opposed to LPS (62).

In summary, respirable crystalline silica activates macrophages toward a pro-inflammatory phenotype, which does not exhibit the same metabolic features of classically LPS-activated macrophages. Upon internalization into phagolysosomes, silica particles regulate the metabolic adaptation of macrophages toward increased uptake of glucose, glycolysis, and lactate secretion, while the mitochondrial respiration becomes sustained only by increased complex II activity, while complex I activity is reduced. Given the role of complex II as a component of both the Krebs cycle and the ETC, its activity becomes a key regulator of the survival of macrophages. The hyperactivation of complex II also modulates the Krebs cycle, where not only succinate and itaconate but all intermediates and amino acids are decreased, probably as a result of high demand and consumption. This new adaptation still provides the source for IL-1β and TNF-α release while suppressing the release of IFN-β.

## 5 Conclusions

In the last decade, enormous progress has been made in understanding the adaptation of metabolic pathways in macrophages and their effect on phenotype and function. However, the schematic illustration that the authors described in this manuscript represents an oversimplification of the complexity of the highly dynamic, tissue-specific, and ontogeny-specific immunometabolic response of macrophages.

In the context of M1 macrophages polarization, many inflammatory signals or pathogens can trigger the pro-inflammatory phenotype, leading to the secretion of pro-inflammatory mediators, even if they express different metabolic signatures. For example, an increased level of succinate and succinate receptors in M1 macrophages underlies the development of many inflammatory diseases, including diabetic retinopathy, diabetic renal disease, hypertension, rheumatoid arthritis, and metabolic dysfunction (46). However, experimental data discussed above have shown that silica-induced macrophage production of pro-inflammatory mediators does not depend on the succinate intracellular accumulation, which is, in fact, depleted in RAW 264.7 macrophages (62). Moreover, in the same environment, alveolar macrophages are polarized toward the pro-fibrotic (M2) phenotype, with minimal glycolysis, augmented oxidative phosphorylation, and fatty acid oxidation (153–155, 157, 158).

Despite the complexity, it is fundamental to understand the intimate connection between immune response and metabolism and how the aberrant metabolic functions are involved in the amplification or inhibition of immune responses. Such understanding can lead to the development of metabolic-targeting drugs especially important for diseases that are still untreatable. For example, metformin, an oral antidiabetic medicine, has already been used to control inflammation through the inhibition of RET-dependent ROS generation (149) and induction of adenosine monophosphate (AMP)-activated protein kinase-dependent fatty acid oxidation (168). It could be possible that a similar approach, targeting the overactivated glycolysis and complex II can rewire the recruited macrophages from a pro-inflammatory toward an anti-inflammatory phenotype to eventually block or delay the progression of chronic inflammation, such as the one observed during the development of silica-induced pulmonary fibrosis.

## Author contributions

The authors (AM and LO) confirm their equal contribution to the paper in the following fields, study conception and design, data collection, analysis and interpretation of results, and draft manuscript preparation. All authors contributed to the article and approved the submitted version.

## Funding

This work was supported by the Department of Health and Human Services (HHS), National Institutes of Health (NIH), National Heart, Lung, and Blood Institute (NHLBI) (grants 1R01HL114795 and 1R01HL110344 to LO), and HHS, NIH, National Institute of Environmental Health Sciences (NIEHS) (grant ES015859 to LO).

## Conflict of interest

The authors declare that the research was conducted in the absence of any commercial or financial relationships that could be construed as a potential conflict of interest.

## Publisher’s note

All claims expressed in this article are solely those of the authors and do not necessarily represent those of their affiliated organizations, or those of the publisher, the editors and the reviewers. Any product that may be evaluated in this article, or claim that may be made by its manufacturer, is not guaranteed or endorsed by the publisher.

## References

[1] Warburg O Wind F Negelein E. The metabolism of tumors in the body. J Gen Physiol (1927) 8(6):519–30. doi: 10.1085/jgp.8.6.519 PMC2140820 19872213

[2] Sbarra AJ Karnovsky ML. The biochemical basis of phagocytosis I Metabolic changes during the ingestion of particles by polymorphonuclear leukocytes. J Biol Chem (1959) 234(6):1355–62. doi: 10.1016/S0021-9258(18)70011-2 13654378

[3] Hard G. Some biochemical aspects of the immune macrophage. Br J Exp pathol (1970) 51(1):97. 5434449 PMC2072214

[4] Newsholme P Gordon S Newsholme EA. Rates of utilization and fates of glucose, glutamine, pyruvate, fatty acids and ketone bodies by mouse macrophages. Biochem J (1987) 242(3):631–6. doi: 10.1042/bj2420631 PMC1147758 3593269

[5] Newsholme P Curi R Gordon S Newsholme EA. Metabolism of glucose, glutamine, long-chain fatty acids and ketone bodies by murine macrophages. Biochem J (1986) 239(1):121–5. doi: 10.1042/bj2390121 PMC1147248 3800971

[6] Murray PJ Wynn TA. Protective and pathogenic functions of macrophage subsets. Nat Rev Immunol (2011) 11(11):723–37. doi: 10.1038/nri3073 PMC3422549 21997792

[7] Byrne AJ Mathie SA Gregory LG Lloyd CM. Pulmonary macrophages: key players in the innate defence of the airways. Thorax (2015) 70(12):1189–96. doi: 10.1136/thoraxjnl-2015-207020 26286722

[8] Ginhoux F Schultze JL Murray PJ Ochando J Biswas SK. New insights into the multidimensional concept of macrophage ontogeny, activation and function. Nat Immunol (2016) 17(1):34–40. doi: 10.1038/ni.3324 26681460

[9] Shapouri-Moghaddam A Mohammadian S Vazini H Taghadosi M Esmaeili SA Mardani F. Macrophage plasticity, polarization, and function in health and disease. J Cell Physiol (2018) 233(9):6425–40. doi: 10.1002/jcp.26429 PMC13482560 29319160

[10] Higgins DM Sanchez-Campillo J Rosas-Taraco AG Higgins JR Lee EJ Orme IM. Relative levels of m-CSF and GM-CSF influence the specific generation of macrophage populations during infection with mycobacterium tuberculosis. J Immunol (2008) 180(7):4892–900. doi: 10.4049/jimmunol.180.7.4892 18354213

[11] Gordon S. Alternative activation of macrophages. Nat Rev Immunol (2003) 3(1):23–35. doi: 10.1038/nri978 12511873

[12] Huang X Xiu H Zhang S Zhang G. The role of macrophages in the pathogenesis of ALI/ARDS. Mediators Inflamm (2018) 2018:1264913. doi: 10.1155/2018/1264913 29950923 PMC5989173

[13] Bellingan GJ. The pulmonary physician in critical care * 6: The pathogenesis of ALI/ARDS. Thorax (2002) 57(6):540–6. doi: 10.1136/thorax.57.6.540 PMC1746355 12037231

[14] De Palma M Lewis CE. Macrophage regulation of tumor responses to anticancer therapies. Cancer Cell (2013) 23(3):277–86. doi: 10.1016/j.ccr.2013.02.013 23518347

[15] Pace JL Russell SW Schreiber RD Altman A Katz DH. Macrophage activation: priming activity from a T-cell hybridoma is attributable to interferon-gamma. Proc Natl Acad Sci (1983) 80(12):3782–6. doi: 10.1073/pnas.80.12.3782 PMC394136 6407020

[16] Nathan CF Murray HW Wiebe ME Rubin BY. Identification of interferon-gamma as the lymphokine that activates human macrophage oxidative metabolism and antimicrobial activity. J Exp Med (1983) 158(3):670–89. doi: 10.1084/jem.158.3.670 PMC2187114 6411853

[17] Mills CD Kincaid K Alt JM Heilman MJ Hill AM. M-1/M-2 macrophages and the Th1/Th2 paradigm. J Immunol (2000) 164(12):6166–73. doi: 10.4049/jimmunol.164.12.6166 10843666

[18] Mantovani A Sica A Sozzani S Allavena P Vecchi A Locati M. The chemokine system in diverse forms of macrophage activation and polarization. Trends Immunol (2004) 25(12):677–86. doi: 10.1016/j.it.2004.09.015 15530839

[19] Orecchioni M Ghosheh Y Pramod AB Ley K. Macrophage polarization: Different gene signatures in M1(LPS+) vs Classically and M2(LPS-) vs. alternatively activated macrophages. Front Immunol (2019) 10:1084. doi: 10.3389/fimmu.2019.01084 31178859 PMC6543837

[20] Stein M Keshav S Harris N Gordon S. Interleukin 4 potently enhances murine macrophage mannose receptor activity: a marker of alternative immunologic macrophage activation. J Exp Med (1992) 176(1):287–92. doi: 10.1084/jem.176.1.287 PMC2119288 1613462

[21] Doyle AG Herbein G Montaner LJ Minty AJ Caput D Ferrara P. Interleukin-13 alters the activation state of murine macrophages *in vitro*: comparison with interleukin-4 and interferon-gamma. Eur J Immunol (1994) 24(6):1441–5. doi: 10.1002/eji.1830240630 7911424

[22] Sawoo R Dey R Ghosh R Bishayi B. TLR4 and TNFR1 blockade dampen M1 macrophage activation and shifts them towards an M2 phenotype. Immunol Res (2021) 69(4):334–51. doi: 10.1007/s12026-021-09209-0 34235623

[23] Scopelliti F Caterina C Valentina D Gianfranco C Concetta M Andrea C. Platelet lysate converts m (IFNγ+ LPS) macrophages in CD206+ TGF-β+ arginase+ M2-like macrophages that affect fibroblast activity and T lymphocyte migration. J Tissue Eng Regen Med (2021) 15(9):788–97. doi: 10.1002/term.3229 34311512

[24] Batista-Gonzalez A Vidal R Criollo A Carreno LJ. New insights on the role of lipid metabolism in the metabolic reprogramming of macrophages. Front Immunol (2019) 10:2993. doi: 10.3389/fimmu.2019.02993 31998297 PMC6966486

[25] Kim TW Shin JS Chung KS Lee YG Baek NI Lee KT. Anti-inflammatory mechanisms of koreanaside a, a lignan isolated from the flower of forsythia koreana, against LPS-induced macrophage activation and DSS-induced colitis mice: The crucial role of AP-1, NF-κB, and JAK/STAT signaling. Cells (2019) 8(10):1163–1181. doi: 10.3390/cells8101163 PMC6829247 31569788

[26] Yunna C Mengru H Lei W Weidong C. Macrophage M1/M2 polarization. Eur J Pharmacol (2020) 877:173090. doi: 10.1016/j.ejphar.2020.173090 32234529

[27] Karshovska E Wei Y Subramanian P Mohibullah R Geißler C Baatsch I. HIF-1α (Hypoxia-inducible factor-1α) promotes macrophage necroptosis by regulating miR-210 and miR-383. Arterioscler thromb Vasc Biol (2020) 40(3):583–96. doi: 10.1161/ATVBAHA.119.313290 31996026

[28] Rius J Guma M Schachtrup C Akassoglou K Zinkernagel AS Nizet V. NF-κB links innate immunity to the hypoxic response through transcriptional regulation of HIF-1α. Nature (2008) 453(7196):807–11. doi: 10.1038/nature06905 PMC2669289 18432192

[29] Wang T Liu H Lian G Zhang S-Y Wang X Jiang C. HIF1α-induced glycolysis metabolism is essential to the activation of inflammatory macrophages. Mediators inflamm (2017) 2017. doi: 10.1155/2017/9029327 PMC5745720 29386753

[30] Yang N Liang Y Yang P Ji F. Propofol suppresses LPS-induced nuclear accumulation of HIF-1α and tumor aggressiveness in non-small cell lung cancer. Oncol Rep (2017) 37(5):2611–9. doi: 10.3892/or.2017.5514 PMC5428906 28426124

[31] Braverman J Stanley SA. Nitric oxide modulates macrophage responses to mycobacterium tuberculosis infection through activation of HIF-1alpha and repression of NF-kappaB. J Immunol (2017) 199(5):1805–16. doi: 10.4049/jimmunol.1700515 PMC5568107 28754681

[32] Dominguez-Gutierrez PR Kusmartsev S Canales BK Khan SR. Calcium oxalate differentiates human monocytes into inflammatory M1 macrophages. Front Immunol (2018) 9:1863. doi: 10.3389/fimmu.2018.01863 30186283 PMC6113402

[33] Rahal OM Wolfe AR Mandal PK Larson R Tin S Jimenez C. Blocking interleukin (IL)4- and IL13-mediated phosphorylation of STAT6 (Tyr641) decreases M2 polarization of macrophages and protects against macrophage-mediated radioresistance of inflammatory breast cancer. Int J Radiat Oncol Biol Phys (2018) 100(4):1034–43. doi: 10.1016/j.ijrobp.2017.11.043 29485045

[34] Rao LZ Wang Y Zhang L Wu G Zhang L Wang FX. IL-24 deficiency protects mice against bleomycin-induced pulmonary fibrosis by repressing IL-4-induced M2 program in macrophages. Cell Death Differ (2021) 28(4):1270–83. doi: 10.1038/s41418-020-00650-6 PMC8027679 33144678

[35] He S Xie L Lu J Sun S. Characteristics and potential role of M2 macrophages in COPD. Int J Chron Obstruct Pulmon Dis (2017) 12:3029–39. doi: 10.2147/COPD.S147144 PMC5655159 29089751

[36] Kim SY Nair MG. Macrophages in wound healing: activation and plasticity. Immunol Cell Biol (2019) 97(3):258–67. doi: 10.1111/imcb.12236 PMC6426672 30746824

[37] Zhu L Fu X Chen X Han X Dong P. M2 macrophages induce EMT through the TGF-β/Smad2 signaling pathway. Cell Biol Int (2017) 41(9):960–8. doi: 10.1002/cbin.10788 28493530

[38] Xu Z-J Gu Y Wang C-Z Jin Y Wen X-M Ma J-C. The M2 macrophage marker CD206: a novel prognostic indicator for acute myeloid leukemia. Oncoimmunology (2020) 9(1):1683347. doi: 10.1080/2162402X.2019.1683347 32002295 PMC6959428

[39] Corliss BA Azimi MS Munson JM Peirce SM Murfee WL. Macrophages: an inflammatory link between angiogenesis and lymphangiogenesis. Microcirculation (2016) 23(2):95–121. doi: 10.1111/micc.12259 26614117 PMC4744134

[40] Becerra-Díaz M Lerner AD Yu DH Thiboutot JP Liu MC Yarmus LB. Sex differences in M2 polarization, chemokine and IL-4 receptors in monocytes and macrophages from asthmatics. Cell Immunol (2021) 360:104252. doi: 10.1016/j.cellimm.2020.104252 33450610 PMC9109226

[41] de Groot AE Pienta KJ. Epigenetic control of macrophage polarization: implications for targeting tumor-associated macrophages. Oncotarget (2018) 9(29):20908. doi: 10.18632/oncotarget.24556 29755698 PMC5945509

[42] Rőszer T. Understanding the mysterious M2 macrophage through activation markers and effector mechanisms. Mediators Inflammation (2015) 2015:1–16. doi: 10.1155/2015/816460 PMC4452191 26089604

[43] Yang R Liao Y Wang L He P Hu Y Yuan D. Exosomes derived from M2b macrophages attenuate DSS-induced colitis. Front Immunol (2019) 10. doi: 10.3389/fimmu.2019.02346 PMC6843072 31749791

[44] Huang X Li Y Fu M Xin H-B. Polarizing macrophages *in vitro* . Macrophages: Springer; (2018) p:119–26. doi: 10.1007/978-1-4939-7837-3_12 PMC8875934 29761394

[45] Nakai K. Multiple roles of macrophage in skin. J Dermatol Sci (2021) 104(1):2–10. doi: 10.1016/j.jdermsci.2021.08.008 34493430

[46] Viola A Munari F Sánchez-Rodríguez R Scolaro T Castegna A. The metabolic signature of macrophage responses. Front Immunol (2019) 10(1462). doi: 10.3389/fimmu.2019.01462 PMC6618143 31333642

[47] Chen Z Dong F Lu J Wei L Tian L Ge H. Polarized M2c macrophages have a promoting effect on the epithelial-to-mesenchymal transition of human renal tubular epithelial cells. Immunobiology (2018) 223(12):826–33. doi: 10.1016/j.imbio.2018.08.008 30172367

[48] Arora S Dev K Agarwal B Das P Syed MA. Macrophages: their role, activation and polarization in pulmonary diseases. Immunobiology (2018) 223(4-5):383–96. doi: 10.1016/j.imbio.2017.11.001 PMC7114886 29146235

[49] Jha AK Huang SC-C Sergushichev A Lampropoulou V Ivanova Y Loginicheva E. Network integration of parallel metabolic and transcriptional data reveals metabolic modules that regulate macrophage polarization. Immunity (2015) 42(3):419–30. doi: 10.1016/j.immuni.2015.02.005 25786174

[50] Meiser J Krämer L Sapcariu SC Battello N Ghelfi J D'Herouel AF. Pro-inflammatory macrophages sustain pyruvate oxidation through pyruvate dehydrogenase for the synthesis of itaconate and to enable cytokine expression. J Biol Chem (2016) 291(8):3932–46. doi: 10.1074/jbc.M115.676817 PMC4759172 26679997

[51] Mills EL Kelly B Logan A Costa ASH Varma M Bryant CE. Succinate dehydrogenase supports metabolic repurposing of mitochondria to drive inflammatory macrophages. Cell (2016) 167(2):457–70.e13. doi: 10.1016/j.cell.2016.08.064 27667687 PMC5863951

[52] Tannahill GM Curtis AM Adamik J Palsson-McDermott EM McGettrick AF Goel G. Succinate is an inflammatory signal that induces IL-1beta through HIF-1alpha. Nature (2013) 496(7444):238–42. doi: 10.1038/nature11986 PMC4031686 23535595

[53] Mills E O'Neill LA. Succinate: a metabolic signal in inflammation. Trends Cell Biol (2014) 24(5):313–20. doi: 10.1016/j.tcb.2013.11.008 24361092

[54] Freemerman AJ Johnson AR Sacks GN Milner JJ Kirk EL Troester MA. Metabolic reprogramming of macrophages: glucose transporter 1 (GLUT1)-mediated glucose metabolism drives a proinflammatory phenotype. J Biol Chem (2014) 289(11):7884–96. doi: 10.1074/jbc.M113.522037 PMC3953299 24492615

[55] Fukuzumi M Shinomiya H Shimizu Y Ohishi K Utsumi S. Endotoxin-induced enhancement of glucose influx into murine peritoneal macrophages *via* GLUT1. Infect immun (1996) 64(1):108–12. doi: 10.1128/iai.64.1.108-112.1996 PMC173734 8557327

[56] Funk JL Feingold KR Moser AH Grunfeld C. Lipopolysaccharide stimulation of RAW 2647 macrophages induces lipid accumulation and foam cell formation. Atherosclerosis (1993) 98(1):67–82. doi: 10.1016/0021-9150(93)90224-I 8457252

[57] Feingold KR Shigenaga JK Kazemi MR McDonald CM Patzek SM Cross AS. Mechanisms of triglyceride accumulation in activated macrophages. J leukocyte Biol (2012) 92(4):829–39. doi: 10.1189/jlb.1111537 PMC3441312 22753953

[58] Mills EL O'Neill LA. Reprogramming mitochondrial metabolism in macrophages as an anti-inflammatory signal. Eur J Immunol (2016) 46(1):13–21. doi: 10.1002/eji.201445427 26643360

[59] Warburn O Dickens F. The metabolism of tumours. Am J Med Sci (1931) 82:123. doi: 10.1097/00000441-193107000-00022

[60] Braverman J Sogi KM Benjamin D Nomura DK Stanley SA. HIF-1alpha is an essential mediator of IFN-gamma-Dependent immunity to mycobacterium tuberculosis. J Immunol (2016) 197(4):1287–97. doi: 10.4049/jimmunol.1600266 PMC4976004 27430718

[61] Hughes MM O'Neill LAJ. Metabolic regulation of NLRP3. Immunol Rev (2018) 281(1):88–98. doi: 10.1111/imr.12608 29247992

[62] Marrocco A Frawley K Pearce LL Peterson J O’Brien JP Mullett SJ. Metabolic adaptation of macrophages as mechanism of defense against crystalline silica. J Immunol (2021) 207(6):1627–40. doi: 10.4049/jimmunol.2000628 PMC8428747 34433619

[63] Talwar H Bouhamdan M Bauerfeld C Talreja J Aoidi R Houde N. MEK2 negatively regulates lipopolysaccharide-mediated IL-1β production through HIF-1α expression. J Immunol (2019) 202(6):1815–25. doi: 10.4049/jimmunol.1801477 PMC6401293 30710049

[64] Talwar H Bauerfeld C Bouhamdan M Farshi P Liu Y Samavati L. MKP-1 negatively regulates LPS-mediated IL-1β production through p38 activation and HIF-1α expression. Cell Signal (2017) 34:1–10. doi: 10.1016/j.cellsig.2017.02.018 28238855 PMC5410178

[65] Palsson-McDermott EM Curtis AM Goel G Lauterbach MA Sheedy FJ Gleeson LE. Pyruvate kinase M2 regulates hif-1α activity and IL-1β induction and is a critical determinant of the warburg effect in LPS-activated macrophages. Cell Metab (2015) 21(1):65–80. doi: 10.1016/j.cmet.2014.12.005 25565206 PMC5198835

[66] Lyou Y Chen G Waterman M. HIF1A can regulate wnt signaling in human colon cancer cells. Cancer Res (2018) 78(13_Supplement):385. doi: 10.1158/1538-7445.AM2018-385

[67] Banoth B Cassel SL. Mitochondria in innate immune signaling. Transl Res (2018) 202:52–68. doi: 10.1016/j.trsl.2018.07.014 30165038 PMC6218307

[68] Palsson-McDermott EM Curtis AM Goel G Lauterbach MAR Sheedy FJ Gleeson LE. Pyruvate kinase M2 regulates hif-1alpha activity and IL-1beta induction and is a critical determinant of the warburg effect in LPS-activated macrophages. Cell Metab (2015) 21(2):347. doi: 0.1016/j.cmet.2014.12.005 29510100 10.1016/j.cmet.2015.01.017

[69] Finucane OM Sugrue J Rubio-Araiz A Guillot-Sestier M-V Lynch MA. The NLRP3 inflammasome modulates glycolysis by increasing PFKFB3 in an IL-1β-dependent manner in macrophages. Sci Rep (2019) 9(1):1–10. doi: 10.1038/s41598-019-40619-1 30858427 PMC6411754

[70] Zhong WJ Yang HH Guan XX Xiong JB Sun CC Zhang CY. Inhibition of glycolysis alleviates lipopolysaccharide-induced acute lung injury in a mouse model. J Cell Physiol (2019) 234(4):4641–54. doi: 10.1002/jcp.27261 30256406

[71] Moon J-S Hisata S Park M-A DeNicola GM Ryter SW Nakahira K. mTORC1-induced HK1-dependent glycolysis regulates NLRP3 inflammasome activation. Cell Rep (2015) 12(1):102–15. doi: 10.1016/j.celrep.2015.05.046 PMC4858438 26119735

[72] Renaudin F Orliaguet L Castelli F Fenaille F Prignon A Alzaid F. Gout and pseudo-gout-related crystals promote GLUT1-mediated glycolysis that governs NLRP3 and interleukin-1β activation on macrophages. Ann Rheum Dis (2020) 79(11):1506–14. doi: 10.1136/annrheumdis-2020-217342 32699039

[73] Van den Bossche J O’Neill LA Menon D. Macrophage immunometabolism: where are we (going)? Trends Immunol (2017) 38(6):395–406. doi: 10.1016/j.it.2017.03.001 28396078

[74] Jin H Zhu Y Wang XD Luo EF Li YP Wang BL. BDNF corrects NLRP3 inflammasome-induced pyroptosis and glucose metabolism reprogramming through KLF2/HK1 pathway in vascular endothelial cells. Cell Signal (2021) 78:109843. doi: 10.1016/j.cellsig.2020.109843 33253911

[75] Michaeloudes C Bhavsar PK Mumby S Xu B Hui CKM Chung KF. Role of metabolic reprogramming in pulmonary innate immunity and its impact on lung diseases. J Innate Immun (2020) 12(1):31–46. doi: 10.1159/000504344 31786568 PMC6959099

[76] Tan HY Wang N Li S Hong M Wang X Feng Y. The reactive oxygen species in macrophage polarization: Reflecting its dual role in progression and treatment of human diseases. Oxid Med Cell Longev (2016) 2016:2795090. doi: 10.1155/2016/2795090 27143992 PMC4837277

[77] West AP Brodsky IE Rahner C Woo DK Erdjument-Bromage H Tempst P. TLR signalling augments macrophage bactericidal activity through mitochondrial ROS. Nature (2011) 472(7344):476–80. doi: 10.1038/nature09973 PMC3460538 21525932

[78] Baardman J Verberk SG Prange KH van Weeghel M van der Velden S Ryan DG. A defective pentose phosphate pathway reduces inflammatory macrophage responses during hypercholesterolemia. Cell Rep (2018) 25(8):2044–52.e5. doi: 10.1016/j.celrep.2018.10.092 30463003

[79] Lee J-H Phelan P Shin M Oh B-C Han X Im S-S. SREBP-1a–stimulated lipid synthesis is required for macrophage phagocytosis downstream of TLR4-directed mTORC1. Proc Natl Acad Sci (2018) 115(52):E12228–E34. doi: 10.1073/pnas.1813458115 PMC6310840 30530672

[80] Williams NC O'Neill LAJ. A role for the Krebs cycle intermediate citrate in metabolic reprogramming in innate immunity and inflammation. Front Immunol (2018) 9:141. doi: 10.3389/fimmu.2018.00141 29459863 PMC5807345

[81] Swain A Bambouskova M Kim H Andhey PS Duncan D Auclair K. Comparative evaluation of itaconate and its derivatives reveals divergent inflammasome and type I interferon regulation in macrophages. Nat Metab (2020) 2(7):594–602. doi: 10.1038/s42255-020-0210-0 32694786 PMC7378276

[82] Murphy MP O'Neill LAJ. Krebs Cycle reimagined: The emerging roles of succinate and itaconate as signal transducers. Cell (2018) 174(4):780–4. doi: 10.1016/j.cell.2018.07.030 30096309

[83] Cordes T Wallace M Michelucci A Divakaruni AS Sapcariu SC Sousa C. Immunoresponsive gene 1 and itaconate inhibit succinate dehydrogenase to modulate intracellular succinate levels. J Biol Chem (2016) 291(27):14274–84. doi: 10.1074/jbc.M115.685792 PMC4933182 27189937

[84] Palmieri EM Gonzalez-Cotto M Baseler WA Davies LC Ghesquière B Maio N. Nitric oxide orchestrates metabolic rewiring in M1 macrophages by targeting aconitase 2 and pyruvate dehydrogenase. Nat Commun (2020) 11(1):1–17. doi: 10.1038/s41467-020-14433-7 32019928 PMC7000728

[85] Santarsiero A Convertini P Todisco S Pierri CL De Grassi A Williams NC. ACLY nuclear translocation in human macrophages drives proinflammatory gene expression by NF-κB acetylation. Cells (2021) 10(11):2962–83. doi: 10.3390/cells10112962 PMC8616537 34831186

[86] Chen Q Cui K Zhao Z Xu X Liu Y Shen Y. LPS stimulation stabilizes HIF-1α by enhancing HIF-1α acetylation *via* the PARP1-SIRT1 and ACLY-Tip60 pathways in macrophages. FASEB J (2022) 36(7):e22418. doi: 10.1096/fj.202200256R 35713568

[87] Saha J Sarkar D Pramanik A Mahanti K Adhikary A Bhattacharyya S. PGE2-HIF1α reciprocal induction regulates migration, phenotypic alteration and immunosuppressive capacity of macrophages in tumor microenvironment. Life Sci (2020) 253:117731. doi: 10.1016/j.lfs.2020.117731 32353431

[88] Koganesawa M Yamaguchi M Samuchiwal SK Balestrieri B. Lipid profile of activated macrophages and contribution of group V phospholipase A(2). Biomolecules (2020) 11(1):338–49. doi: 10.3390/biom11010025 PMC7823364 33383652

[89] Galvan-Pena S Carroll RG Newman C Hinchy EC Palsson-McDermott E Robinson EK. Malonylation of GAPDH is an inflammatory signal in macrophages. Nat Commun (2019) 10(1):338. doi: 10.1038/s41467-018-08187-6 30659183 PMC6338787

[90] Wu R Chen F Wang N Tang D Kang R. ACOD1 in immunometabolism and disease. Cell Mol Immunol (2020) 17(8):822–33. doi: 10.1038/s41423-020-0489-5 PMC7395145 32601305

[91] Gidon A Louet C Røst LM Bruheim P Flo TH. The tumor necrosis factor alpha and interleukin 6 auto-paracrine signaling loop controls mycobacterium avium infection *via* induction of IRF1/IRG1 in human primary macrophages. mBio (2021) 12(5):e0212121. doi: 10.1128/mBio.02121-21 34607464 PMC8546851

[92] Li Y Gong W Li W Liu P Liu J Jiang H. The IRG1-itaconate axis: A regulatory hub for immunity and metabolism in macrophages. Int Rev Immunol (2022) 1–15. doi: 10.1080/08830185.2022.2067153 35468044

[93] Domínguez-Andrés J Novakovic B Li Y Scicluna BP Gresnigt MS Arts RJW. The itaconate pathway is a central regulatory node linking innate immune tolerance and trained immunity. Cell Metab (2019) 29(1):211–20.e5. doi: 10.1016/j.cmet.2018.09.003 30293776

[94] Berg IA Filatova LV Ivanovsky RN. Inhibition of acetate and propionate assimilation by itaconate *via* propionyl-CoA carboxylase in isocitrate lyase-negative purple bacterium rhodospirillum rubrum. FEMS Microbiol letters. (2002) 216(1):49–54. doi: 10.1111/j.1574-6968.2002.tb11413.x 12423751

[95] Michelucci A Cordes T Ghelfi J Pailot A Reiling N Goldmann O. Immune-responsive gene 1 protein links metabolism to immunity by catalyzing itaconic acid production. Proc Natl Acad Sci U S A. (2013) 110(19):7820–5. doi: 10.1073/pnas.1218599110 PMC3651434 23610393

[96] Naujoks J Tabeling C Dill BD Hoffmann C Brown AS Kunze M. IFNs modify the proteome of legionella-containing vacuoles and restrict infection *via* IRG1-derived itaconic acid. PloS pathogens. (2016) 12(2):e1005408. doi: 10.1371/journal.ppat.1005408 26829557 PMC4734697

[97] Mills EL Ryan DG Prag HA Dikovskaya D Menon D Zaslona Z. Itaconate is an anti-inflammatory metabolite that activates Nrf2 *via* alkylation of KEAP1. Nature (2018) 556(7699):113–7. doi: 10.1038/nature25986 PMC6047741 29590092

[98] Ahmed SM Luo L Namani A Wang XJ Tang X. Nrf2 signaling pathway: Pivotal roles in inflammation. Biochim Biophys Acta Mol Basis Dis (2017) 1863(2):585–97. doi: 10.1016/j.bbadis.2016.11.005 27825853

[99] Kobayashi E Suzuki T Funayama R Nagashima T Hayashi M Sekine H. Nrf2 suppresses macrophage inflammatory response by blocking proinflammatory cytokine transcription. Nat Commun (2016) 7:11624. doi: 10.1038/ncomms11624 27211851 PMC4879264

[100] O'Neill LAJ Artyomov MN. Itaconate: the poster child of metabolic reprogramming in macrophage function. Nat Rev Immunol (2019) 19(5):273–81. doi: 10.1038/s41577-019-0128-5 30705422

[101] Hooftman A Angiari S Hester S Corcoran SE Runtsch MC Ling C. The immunomodulatory metabolite itaconate modifies NLRP3 and inhibits inflammasome activation. Cell Metab (2020) 32(3):468–78.e7. doi: 10.1016/j.cmet.2020.07.016 32791101 PMC7422798

[102] Yi Z Deng M Scott MJ Fu G Loughran PA Lei Z. Immune-responsive gene 1/Itaconate activates nuclear factor erythroid 2-related factor 2 in hepatocytes to protect against liver ischemia-reperfusion injury. Hepatology (2020) 72(4):1394–411. doi: 10.1002/hep.31147 PMC7702080 31997373

[103] Lampropoulou V Sergushichev A Bambouskova M Nair S Vincent EE Loginicheva E. Itaconate links inhibition of succinate dehydrogenase with macrophage metabolic remodeling and regulation of inflammation. Cell Metab (2016) 24(1):158–66. doi: 10.1016/j.cmet.2016.06.004 PMC5108454 27374498

[104] Macias-Ceja DC Ortiz-Masiá D Salvador P Gisbert-Ferrándiz L Hernández C Hausmann M. Succinate receptor mediates intestinal inflammation and fibrosis. Mucosal Immunol (2019) 12(1):178–87. doi: 10.1038/s41385-018-0087-3 30279517

[105] Scialò F Fernández-Ayala DJ Sanz A. Role of mitochondrial reverse electron transport in ROS signaling: Potential roles in health and disease. Front Physiol (2017) 8:428. doi: 10.3389/fphys.2017.00428 28701960 PMC5486155

[106] Roca FJ Whitworth LJ Prag HA Murphy MP Ramakrishnan L. Tumor necrosis factor induces pathogenic mitochondrial ROS in tuberculosis through reverse electron transport. Science (2022) 376(6600):eabh2841. doi: 10.1126/science.abh2841 35737799 PMC7612974

[107] Hatinguais R Pradhan A Brown GD Brown AJP Warris A Shekhova E. Mitochondrial reactive oxygen species regulate immune responses of macrophages to aspergillus fumigatus. Front Immunol (2021) 12:641495. doi: 10.3389/fimmu.2021.641495 33841423 PMC8026890

[108] Knight M Braverman J Asfaha K Gronert K Stanley S. Lipid droplet formation in mycobacterium tuberculosis infected macrophages requires IFN-gamma/HIF-1alpha signaling and supports host defense. PloS Pathog (2018) 14(1):e1006874. doi: 10.1371/journal.ppat.1006874 29370315 PMC5800697

[109] Iommarini L Porcelli AM Gasparre G Kurelac I. Non-canonical mechanisms regulating hypoxia-inducible factor 1 alpha in cancer. Front Oncol (2017) 7:286. doi: 10.3389/fonc.2017.00286 29230384 PMC5711814

[110] Zuo H Wan Y. Metabolic reprogramming in mitochondria of myeloid cells. Cells (2019) 9(1):5–22. doi: 10.3390/cells9010005 PMC7017304 31861356

[111] Gomes MTR Guimaraes ES Marinho FV Macedo I Aguiar E Barber GN. STING regulates metabolic reprogramming in macrophages *via* HIF-1alpha during brucella infection. PloS Pathog (2021) 17(5):e1009597. doi: 10.1371/journal.ppat.1009597 33989349 PMC8153530

[112] Malkov MI Lee CT Taylor CT. Regulation of the hypoxia-inducible factor (HIF) by pro-inflammatory cytokines. Cells (2021) 10(9):2340–55. doi: 10.3390/cells10092340 PMC8466990 34571989

[113] O'Neill LAJ. The hunger games: Salmonella, anorexia, and NLRP3. Cell Metab (2017) 25(2):225–6. doi: 10.1016/j.cmet.2017.01.015 28178561

[114] O'Neill LAJ Zaslona Z. Macrophages remember cheeseburgers and promote inflammation *via* NLRP3. Trends Mol Med (2018) 24(4):335–7. doi: 10.1016/j.molmed.2018.02.005 29483038

[115] Mills EL Kelly B O'Neill LAJ. Mitochondria are the powerhouses of immunity. Nat Immunol (2017) 18(5):488–98. doi: 10.1038/ni.3704 28418387

[116] Wei Z Liu X Cheng C Yu W Yi P. Metabolism of amino acids in cancer. Front Cell Dev Biol (2021) 8. doi: 10.3389/fcell.2020.603837 PMC7835483 33511116

[117] Fall F Lamy E Brollo M Naline E Lenuzza N Thévenot E. Metabolic reprograming of LPS-stimulated human lung macrophages involves tryptophan metabolism and the aspartate-arginosuccinate shunt. PloS One (2020) 15(4):e0230813. doi: 10.1371/journal.pone.0230813 32267860 PMC7141605

[118] Liu PS Wang H Li X Chao T Teav T Christen S. α-ketoglutarate orchestrates macrophage activation through metabolic and epigenetic reprogramming. Nat Immunol (2017) 18(9):985–94. doi: 10.1038/ni.3796 28714978

[119] Xia Y He F Wu X Tan B Chen S Liao Y. GABA transporter sustains IL-1β production in macrophages. Sci Adv (2021) 7(15):9274–92. doi: 10.1126/sciadv.abe9274 PMC8026138 33827820

[120] Duanmu Q Tan B Wang J Huang B Li J Kang M. The amino acids sensing and utilization in response to dietary aromatic amino acid supplementation in LPS-induced inflammation piglet model. Front Nutr (2021) 8:819835. doi: 10.3389/fnut.2021.819835 35111801 PMC8801454

[121] Kelly B O'Neill LA. Metabolic reprogramming in macrophages and dendritic cells in innate immunity. Cell Res (2015) 25(7):771–84. doi: 10.1038/cr.2015.68 PMC4493277 26045163

[122] Palmieri EM McGinity C Wink DA McVicar DW. Nitric oxide in macrophage immunometabolism: hiding in plain sight. Metabolites (2020) 10(11):429. doi: 10.3390/metabo10110429 PMC7693038 33114647

[123] Vishwakarma A Kumari A Mur LA Gupta KJ. A discrete role for alternative oxidase under hypoxia to increase nitric oxide and drive energy production. Free Radical Biol Med (2018) 122:40–51. doi: 10.1016/j.freeradbiomed.2018.03.045 29604396

[124] Carneiro FRG Lepelley A Seeley JJ Hayden MS Ghosh S. An essential role for ECSIT in mitochondrial complex I assembly and mitophagy in macrophages. Cell Rep (2018) 22(10):2654–66. doi: 10.1016/j.celrep.2018.02.051 PMC5909989 29514094

[125] Garaude J Acin-Perez R Martinez-Cano S Enamorado M Ugolini M Nistal-Villan E. Mitochondrial respiratory-chain adaptations in macrophages contribute to antibacterial host defense. Nat Immunol (2016) 17(9):1037–45. doi: 10.1038/ni.3509 PMC4994870 27348412

[126] Holmbeck MA Shadel GS. Mitochondria provide a 'complex' solution to a bacterial problem. Nat Immunol (2016) 17(9):1009–10. doi: 10.1038/ni.3534 27540983

[127] Gottesfeld P Reid M Goosby E. Preventing tuberculosis among high-risk workers. Lancet Global Health (2018) 6(12):e1274–e5. doi: 10.1016/S2214-109X(18)30313-9 30262448

[128] Anderson SE Shane H Long C Marrocco A Lukomska E Roberts JR. Biological effects of inhaled hydraulic fracturing sand dust. VIII. immunotoxicity. Toxicol Appl Pharmacol (2020) 408:115256. doi: 10.1016/j.taap.2020.115256 33007384 PMC7796771

[129] Leung CC Yu ITS Chen W. Silicosis. Lancet (2012) 379(9830):2008–18. doi: 10.1016/S0140-6736(12)60235-9 22534002

[130] Di Giuseppe M Gambelli F Hoyle GW Lungarella G Studer SM Richards T. Systemic inhibition of NF-kappaB activation protects from silicosis. PloS One (2009) 4(5):e5689. doi: 10.1371/journal.pone.0005689 19479048 PMC2682759

[131] WHO. Elimination of silicosis, GOHNET newsletter (2007). Available at: https://www.who.int/occupational_health/publications/newsletter/gohnet12e.pdf?ua=1.

[132] Society AT. Adverse effects on crystalline silica exposure. Am J Crit Care Med (1997) 155:761–5. doi: 10.1164/ajrccm.155.2.9032226 9032226

[133] Walters EH Shukla SD. Silicosis: Pathogenesis and utility of animal models of disease. Allergy (2021) 76(10):3241–2. doi: 10.1111/all.14880 34596272

[134] Barnes H Goh NSL Leong TL Hoy R. Silica-associated lung disease: An old-world exposure in modern industries. Respirology (2019) 24(12):1165–75. doi: 10.1111/resp.13695 31517432

[135] Tan S Chen S. Macrophage autophagy and silicosis: Current perspective and latest insights. Int J Mol Sci (2021) 22(1):453–66. doi: 10.3390/ijms22010453 PMC7795780 33466366

[136] Adamcakova J Mokra D. New insights into pathomechanisms and treatment possibilities for lung silicosis. Int J Mol Sci (2021) 22(8):4162–84. doi: 10.3390/ijms22084162 PMC8072896 33920534

[137] Hou X Summer R Chen Z Tian Y Ma J Cui J. Lipid uptake by alveolar macrophages drives fibrotic responses to silica dust. Sci Rep (2019) 9(1):399. doi: 10.1038/s41598-018-36875-2 30674959 PMC6344530

[138] Hirano S Zhou Q Furuyama A Kanno S. Differential regulation of IL-1β and IL-6 release in murine macrophages. Inflammation (2017) 40(6):1933–43. doi: 10.1007/s10753-017-0634-1 28766178

[139] Cassel SL Eisenbarth SC Iyer SS Sadler JJ Colegio OR Tephly LA. The Nalp3 inflammasome is essential for the development of silicosis. Proc Natl Acad Sci U S A. (2008) 105(26):9035–40. doi: 10.1073/pnas.0803933105 PMC2449360 18577586

[140] Lam M Mansell A Tate MD. Another one fights the dust-targeting the NLRP3 inflammasome for the treatment of silicosis. Am J Respir Cell Mol Biol (2022) 66:601–11. doi: 10.1165/rcmb.2021-0545TR 35290170

[141] Liu X Lu B Fu J Zhu X Song E Song Y. Amorphous silica nanoparticles induce inflammation via activation of NLRP3 inflammasome and HMGB1/TLR4/MYD88/NF-kb signaling pathway in HUVEC cells. J Hazard Mater (2021) 404(Pt B):124050. doi: 10.1016/j.jhazmat.2020.124050 33053467

[142] Costantini LM Gilberti RM Knecht DA. The phagocytosis and toxicity of amorphous silica. PloS One (2011) 6(2):e14647. doi: 10.1371/journal.pone.0014647 21311600 PMC3032735

[143] Dostert C Pétrilli V Van Bruggen R Steele C Mossman BT Tschopp J. Innate immune activation through Nalp3 inflammasome sensing of asbestos and silica. Science (2008) 320(5876):674–7. doi: 10.1126/science.1156995 PMC2396588 18403674

[144] Beamer CA Holian A. Silica suppresses toll-like receptor ligand-induced dendritic cell activation. FASEB J (2008) 22(6):2053–63. doi: 10.1096/fj.07-095299 18180331

[145] Hornung V Bauernfeind F Halle A Samstad EO Kono H Rock KL. Silica crystals and aluminum salts activate the NALP3 inflammasome through phagosomal destabilization. Nat Immunol (2008) 9(8):847–56. doi: 10.1038/ni.1631 PMC2834784 18604214

[146] Fazzi F Njah J Di Giuseppe M Winnica DE Go K Sala E. TNFR1/phox interaction and TNFR1 mitochondrial translocation thwart silica-induced pulmonary fibrosis. J Immunol (2014) 192(8):3837–46. doi: 10.4049/jimmunol.1103516 PMC3977215 24623132

[147] Mischler SE Cauda EG Di Giuseppe M McWilliams LJ St Croix C Sun M. Differential activation of RAW 264.7 macrophages by size-segregated crystalline silica. J Occup Med Toxicol (2016) 11:57. doi: 10.1186/s12995-016-0145-2 28018477 PMC5159951

[148] Fubini B Hubbard A. Reactive oxygen species (ROS) and reactive nitrogen species (RNS) generation by silica in inflammation and fibrosis. Free Radic Biol Med (2003) 34(12):1507–16. doi: 10.1016/S0891-5849(03)00149-7 12788471

[149] Kelly B Tannahill GM Murphy MP O'Neill LA. Metformin inhibits the production of reactive oxygen species from NADH:Ubiquinone oxidoreductase to limit induction of interleukin-1beta (IL-1beta) and boosts interleukin-10 (IL-10) in lipopolysaccharide (LPS)-activated macrophages. J Biol Chem (2015) 290(33):20348–59. doi: 10.1074/jbc.M115.662114 PMC4536441 26152715

[150] Gambelli F Di P Niu X Friedman M Hammond T Riches DW. Phosphorylation of tumor necrosis factor receptor 1 (p55) protects macrophages from silica-induced apoptosis. J Biol Chem (2004) 279(3):2020–9. doi: 10.1074/jbc.M309763200 14570868

[151] Zhu Y Yao J Duan Y Xu H Cheng Q Gao X. Protein expression profile in rat silicosis model reveals upregulation of PTPN2 and its inhibitory effect on epithelial-mesenchymal transition by dephosphorylation of STAT3. Int J Mol Sci (2020) 21(4):1189. doi: 10.3390/ijms21041189 PMC7072761 32054021

[152] Gozal E Ortiz LA Zou X Burow ME Lasky JA Friedman M. Silica-induced apoptosis in murine macrophage: involvement of tumor necrosis factor-α and nuclear factor-κ b activation. Am J Respir Cell Mol Biol (2002) 27(1):91–8. doi: 10.1165/ajrcmb.27.1.4790 12091251

[153] Huang L Nazarova EV Tan S Liu Y Russell DG. Growth of mycobacterium tuberculosis *in vivo* segregates with host macrophage metabolism and ontogeny. J Exp Med (2018) 215(4):1135–52. doi: 10.1084/jem.20172020 PMC5881470 29500179

[154] Mould KJ Barthel L Mohning MP Thomas SM McCubbrey AL Danhorn T. Cell origin dictates programming of resident versus recruited macrophages during acute lung injury. Am J Respir Cell Mol Biol (2017) 57(3):294–306. doi: 10.1165/rcmb.2017-0061OC 28421818 PMC5625228

[155] Pisu D Huang L Grenier JK Russell DG. Dual RNA-seq of mtb-infected macrophages *in vivo* reveals ontologically distinct host-pathogen interactions. Cell Rep (2020) 30(2):335–50.e4. doi: 10.1016/j.celrep.2019.12.033 31940480 PMC7032562

[156] Zhao Y Hao C Bao L Wang D Li Y Qu Y. Silica particles disorganize the polarization of pulmonary macrophages in mice. Ecotoxicol Environ safety. (2020) 193:110364. doi: 10.1016/j.ecoenv.2020.110364 32114243

[157] Woods PS Kimmig LM Meliton AY Sun KA Tian Y O’Leary EM. Tissue-resident alveolar macrophages do not rely on glycolysis for LPS-induced inflammation. Am J Respir Cell Mol Biol (2020) 62(2):243–55. doi: 10.1165/rcmb.2019-0244OC PMC6993551 31469581

[158] Khaing P Summer R. Maxed out on glycolysis: alveolar macrophages rely on oxidative phosphorylation for cytokine production. Am J Respir Cell Mol Biol (2020) 62(2):139–40. doi: 10.1165/rcmb.2019-0329ED PMC6993550 31560565

[159] Saborano R Wongpinyochit T Totten JD Johnston BF Seib FP Duarte IF. Metabolic reprogramming of macrophages exposed to silk, poly(lactic-co-glycolic acid), and silica nanoparticles. Adv Healthc Mater (2017) 6(14):1–13. doi: 10.1002/adhm.201601240 28544603

[160] McElvaney OJ Zaslona Z Becker-Flegler K Palsson-McDermott EM Boland F Gunaratnam C. Specific inhibition of the NLRP3 inflammasome as an antiinflammatory strategy in cystic fibrosis. Am J Respir Crit Care Med (2019) 200(11):1381–91. doi: 10.1164/rccm.201905-1013OC 31454256

[161] Yang M Wang D Gan S Wang B Yu L Xie Y. Triiodothyronine ameliorates silica-induced pulmonary inflammation and fibrosis in mice. Sci Total Environ (2021) 790:148041. doi: 10.1016/j.scitotenv.2021.148041 34090168

[162] Mao N Yang H Yin J Li Y Jin F Li T. Glycolytic reprogramming in silica-induced lung macrophages and silicosis reversed by Ac-SDKP treatment. Int J Mol Sci (2021) 22(18):10063. doi: 10.3390/ijms221810063 34576239 PMC8465686

[163] Cai W Zhang B Li T Jin F Li Y Xu H. Transcriptomic analysis identifies upregulation of secreted phosphoprotein 1 in silicotic rats. Exp Ther Med (2021) 21(6):579. doi: 10.3892/etm.2021.10011 33850551 PMC8027763

[164] Nie W Lan T Yuan X Luo M Shen G Yu J. Crystalline silica induces macrophage necrosis and causes subsequent acute pulmonary neutrophilic inflammation. Cell Biol Toxicol (2021) 38(4):591–609. doi: 10.1007/s10565-021-09620-1 34170461

[165] Tan S Yang S Chen M Wang Y Zhu L Sun Z. Lipopolysaccharides promote pulmonary fibrosis in silicosis through the aggravation of apoptosis and inflammation in alveolar macrophages. Open Life Sci (2020) 15(1):598–605. doi: 10.1515/biol-2020-0061 33817248 PMC7874552

[166] Wu R Hogberg J Adner M Ramos-Ramirez P Stenius U Zheng H. Crystalline silica particles cause rapid NLRP3-dependent mitochondrial depolarization and DNA damage in airway epithelial cells. Part Fibre Toxicol (2020) 17(1):39. doi: 10.1186/s12989-020-00370-2 32778128 PMC7418441

[167] Srivastava KD Rom WN Jagirdar J Yie T-A Gordon T Tchou-Wong K-M. Crucial role of interleukin-1 β and nitric oxide synthase in silica-induced inflammation and apoptosis in mice. Am J Respir Crit Care Med (2002) 165(4):527–33. doi: 10.1164/ajrccm.165.4.2106009 11850347

[168] Tokubuchi I Tajiri Y Iwata S Hara K Wada N Hashinaga T. Beneficial effects of metformin on energy metabolism and visceral fat volume through a possible mechanism of fatty acid oxidation in human subjects and rats. PloS One (2017) 12(2):e0171293. doi: 10.1371/journal.pone.0171293 28158227 PMC5291441

